# Impact of the introduction of high-speed rail on the income gap between urban and rural residents

**DOI:** 10.1371/journal.pone.0292105

**Published:** 2023-11-27

**Authors:** Dapeng Sun, Xu Zhao, Guangli Zhang, Pengyu Chen

**Affiliations:** 1 School of Marxism, Dalian Maritime University, Dalian, Liaoning, China; 2 Surrey International Institute, Dongbei University of Finance and Economics, Dalian, Liaoning, China; 3 School of Economics, Dongbei University of Finance and Economics, Dalian, Liaoning, China; National University of Sciences and Technology, PAKISTAN

## Abstract

The unprecedented expansion and development of high-speed rail (HSR) in China provides a unique opportunity and a new way of thinking for addressing the problem of urban-rural wealth disparities. In this paper, I examine the impact of the introduction of HSRs on the income disparity between urban and rural residents in China. Using panel data from 285 prefecture-level cities from 2004 to 2018, in this paper I employ the double-difference method to assess the impact of HSR on the income gap between urban and rural populations and the mechanism of its action; furthermore, I explore the influence of HSR on urban residents’ per capita disposable income and rural residents’ per capita net income, as well as the impact of HSR on the flow of elements such as labor and capital. My research findings reveal that the introduction of HSR has greatly widened the income gap between urban and rural residents; however, there is heterogeneity between different East, Central, and West regions, as well as between different levels of cities. A further mechanism study finds that HSR lowers farmers’ per capita net income, raises urban residents’ per capita disposable income, and widens the urban/rural income gap via mechanisms such as facilitating the interregional mobility of labor and capital factors. Therefore, it is necessary to comprehensively assess the economic effects brought about by HSR, strengthen the construction of urban-rural transport networks, and improve support for rural areas, so as to promote the coordinated development of inter-regional and urban-rural areas.

## 1 Introduction

### 1.1 Research background

Since the reform and opening up of China, the country’s economy has been developing rapidly, and the people’s economic and living standards have improved in leaps and bounds, with an unprecedented increase in comprehensive national power. However, while China has made brilliant achievements since its reform and opening up, many contradictions and discordant factors have also emerged; behind rapid economic growth, the excessive and difficult-to-reconcile gap between urban and rural incomes has always been a problem that is hard to ignore, and China’s regional and urban-rural development gaps are still the main destabilizing factors existing in Chinese society at present. Since the beginning of the 1990s, there has been a widening trend in the urban-rural income gap in China, and according to information released by the National Bureau of Statistics, the ratio of China’s urban-rural income gap rose from 2.57 in 1978 to 3.3 in 2010, which was much higher than the world average of the urban-rural income gap at that time, which stood at 1.8. Despite the fact that the urban-rural income gap has been narrowing year by year since 2010, the problem is still relatively serious when compared with that of developed countries, and has become an important social contradiction and a difficult problem that needs to be solved urgently.

By constructing high-speed rail (HSR) networks throughout the whole nation, China has made tremendous progress in recent years in developing its HSR infrastructure. HSR reduces travel times and distances, lowers the cost of delivery and transportation, and promotes the movement of industrial inputs across the region. During the period from the initial operation of HSR on 1 August 2008 to 11 July 2016, China’s HSR has serviced more than 5 billion passengers cumulatively, and in 2020, the passenger turnover of HSR will account for 25.2 per cent of total social passenger traffic, cargo transport volume will exceed 3.58 billion tons, and the share of the total society in freight traffic will increase to 9.6 per cent.

Chinese researchers began investigating whether there was a correlation between HSR and the advancement of local economies as the technology for HSR continued to progress. Academics now consider that HSR may be able to better fulfill the requirements of the flow of production factors, which would subsequently boost growth in the economy and alter China’s regional economic development pattern. Given the rapid adoption of HSR and the significant wealth disparity between urban and rural inhabitants in China, is there a connection between the introduction of HSR and the expansion or contraction of the economic gap between urban and rural areas in the country? How does the functioning of the HSR system affect the wealth difference between urban and rural areas? If we examine these concerns in more detail and come up with some workable solutions, we will be able to see how essential HSR is to economic growth, so as to clearly assess the advantages and shortcomings, opportunities, and challenges brought about by the HSR economy, and further provide corresponding policy suggestions for narrowing the income gap between urban and rural areas and promoting the development of the regional economy. It is of practical significance and academic value to begin our research on this issue from this starting point.

### 1.2 Significance of the research

When studying whether the development of HSR contributes to the narrowing of the urban-rural income gap, it will be beneficial to analyze and verify the economic effect of HSR from a unique perspective, and provide an entry point for solving the problem of China’s urban-rural income gap and even China’s smooth economic development, and I hope to gain some useful inspiration and new exploration ideas as a result. Although many scholars have paid attention to the research on the impact of infrastructure on economic growth, there is relatively little research on HSR, the urban-rural income gap, and regional economic development. As a new transport mode in recent years, HSR, although starting late, has developed faster than other types transport infrastructure such as highways, which is due to its fast speed, large capacity, and high degree of convenience. It is precisely because advantages of HSR are incomparable to other types of transport infrastructure that the study of the economic effect of HSR and its impact on the urban-rural income gap has unique theoretical significance and practical value.

(1) Theoretical significance: In the previous studies on the influencing factors of the urban-rural income gap, researchers seldom pay attention to the influence of transport infrastructure and means of transport, and mostly focus on traditional and relatively easy-to-measure indicators, such as GDP per capita, industrial structure, financial expenditure, etc.; the research that exists lacks depth. In this paper, I take HSR, which is a newly-emergent transport infrastructure with strong development momentum in recent years, as an entry point to study its impact on the urban-rural income gap, and try to clarify the mechanism of HSR’s impact on this gap, which can enrich existing research to a certain extent, and has a certain degree of theoretical significance.

(2) Practical value: In light of today’s steady economic development, it has become imperative to close the income gap between urban and rural areas. Through research on the urban-rural income gap, we learn that it is normal to have an appropriate income gap between urban and rural residents, and the existence of the gap will generate the free flow of labor and capital, which is also required by the socialist market mechanism. A certain income gap can drive the enthusiasm of laborers in production and work, and bring motivation and innovation; but an income gap that is too wide will harm the long-term development of socialism, which will not only hinder rapid economic growth, but also be detrimental to the long-term stability of society. HSR is fast, safe, and reliable, which can relieve the pressure on the transport of people and goods, stimulate regional economic development along its route, promote industrial transformation and upgrading, and create employment opportunities. The economic benefits of HSR are quite prominent, so the study of the impact of HSR on the urban-rural income gap has a considerable degree of practical value for the coordinated development of regional economies and the construction of a harmonious socialist society.

### 1.3 Innovation points

(1) There is a lot of research on the impact of urban-rural residents’ income gap, but there is less research from the perspective of infrastructure, especially from the perspective of HSR, and no consistent conclusion has yet been reached. In this paper I take the HSR as an entry point to study its impact on the urban-rural income gap, which enriches the concept of the impact of transport infrastructure on the economy.

(2) In this paper I not only analyze the impact of HSR on the urban-rural income gap, but also the impact on urban residents’ income and rural residents’ income, and examine the mechanism of HSR’s impact on urban-rural residents’ income gap from the perspective of factor mobility (factors such as labor force and capital), which increases the depth provided by previous research.

(3) In terms of research methodology, taking the introduction of HSR as a perspective, treating "HSR" as a quasi-natural experiment, and applying the multi-period double-difference method to carry out the research, to a certain extent, it is possible to overcome the problem of endogeneity, and it is helpful to improve the accuracy of the research results by selecting more comprehensive data for analysis.

## 2 Literature review

### 2.1 Research on the impact of the urban-rural income gap

Since the 1980s, the discrepancy in wealth between China’s urban and rural inhabitants has widened dramatically and is much greater than the global average. At the same time, there were stark disparities in the income levels between China’s urban and rural communities (Haiming et al., 2015) [1]. The exogenous urban-rural income gap hypothesis and endogenous urban-rural income gap theory are the two fundamental theories that aim to explain the elements responsible for the economic discrepancy between urban and rural regions. The bulk of the emphasis dedicated to urban-orientation policy is concentrated on the exogenous urban-rural income gap theory. According to Yang (1999) [2] and Hannum and Park (2002) [3], action taken by the government to encourage the expansion of cities has resulted in greater disparity between urban and rural regions, including aspects such as the imbalanced growth of labour-intensive sectors due to the strategy of prioritising the development of heavy industries (Binkai and Yifu, 2013) [4]. Income disparities were exacerbated by the development strategy’s emphasis on heavy industries (Jing et al., 2018) [5]. Three of the most essential components of the endogenous urban-rural income gap hypothesis are urbanization, labor mobility, and financial resources. Urban-rural regions have a dual structure. First, research by Sicular et al. (2007) [6], Haiyuan et al. (2013) [7], and others has shown that the household registration system adds to the income discrepancy between urban and rural regions. The second factor is a region’s urbanization. Bangyuan and Liang (2014) [8] drew the conclusion that urbanization widened the urban-rural income difference after evaluating the dynamic relationships between industrialization, urbanization, and the inequality they create. Ziye et al. (2016) [9], who evaluated panel data from 30 provinces and cities between 2003 and 2013, found that urbanization exhibited an "inverted U-shaped pattern" that expanded the income difference between urban and rural regions. This increased the discrepancy between the living conditions of the two types of community. Another topic to think about is the issue of money. According to a study done in 2011 by Zhiqiang [10] and his colleagues, there exists a direct connection between the degree of financial development and the economic inequality between urban and rural areas. Zeyuan et al. (2021) [11] hypothesized that by bringing urban and rural households’ financial habits closer together, it would be possible to limit the widening of the economic gap between them. Human capital should be included as the fourth component. One aspect that led to the disparity in economic well-being between the two kinds of localities was the uneven allocation of educational resources between urban and rural areas (Binkai et al., 2010) [12]. Because of the disparity in educational possibilities that exists between urban and rural areas, there will necessarily be a bigger economic difference between the two types of location (Wei et al., 2015) [13].

### 2.2 Research on HSR

HSR is fast, safe, and reliable, which not only has a profound impact on the economy and society, but also becomes an important symbol of modern civilization. Most of the existing studies on HSR focus on the causes of HSR development and a discussion of its effect on regional economic growth, scientific and technological innovation, and enterprise competitiveness.

(1) From the viewpoint of the motivation of HSR development, foreign scholars have pointed out that the direct motivation of the country’s vigorous development of HSR does not come from the economic effect of HSR, and the country attaches more importance to the cost of constructing HSR rather than its economic benefits. Therefore, many scholars mostly study the causes of HSR development from its externalities. Although the cost of HSR is high, it has unique advantages over other transportation infrastructure, such as its higher speed, environmental protection, and energy saving, and to a certain extent, it can alleviate the problem of traffic congestion.

Some commentators claim that the benefits of HSR for the economy were not the main factors fueling the growth of HSR in China, and the expense of building HSR in the majority of other nations was often higher than their benefits (Albalate et al., 2012) [14]. Givoni (2006) [15] discovered that other transportation facilities’ traffic demands will be shared by the development of Japan’s high-speed Shinkansen train. Additionally, HSR may significantly lower carbon dioxide emissions and pollution levels that cause urban haze (Jianming et al., 2020) [16]. Furthermore, Nakagawa and Hatoko (2007) [17] discovered that, despite the expense, building an HSR would be crucial owing to the advantages it would bring to the environment. Cvetanovic (2013) argues that HSR construction is based on the strategic importance of HSR to the country, and considering its future value, its solving of transportation problems, and its energy saving and emission reduction, it is still necessary to increase investment in HSR construction despite the high cost of HSR construction.

(2) Regarding the intrinsic mechanism of the HSR economy, Xiaoyan (2018) [18] asserts that the only way to determine how the high-speed train sector functions internally is to comprehend its economic features. The two primary concepts are the scale economics theory of the transportation sector and the value theory of travel time reduction. The value theory of saving travel time postulates that time has the same value for travelers, and if travelers save on travel time and use it for other productive activities, they can create more value to satisfy their own needs and the needs of society; and since HSRs run faster than ordinary trains, they have more value in saving passengers’ travel time, which will result in a greater economic effect. Wu et al. (2014) use the value of travel time as the basis to study whether HSR helps to solve the difficulties of China’s railroad problems, and conclude that the formulation of HSR policy should be adapted to the local conditions, and rationally formulated according to the regional location. Tianjun and Xueyun (1999) analyzed the value of travel time savings of the Beijing-Shanghai HSR and found that the value of travel time per unit along the Beijing-Shanghai route was greater than twice the national average, confirming that the construction of HSR can create greater value by saving travelers’ time. Wei (2000) analyzed the value of travelers’ travel time in regard to highway construction and established a model of the value of travelers’ free choice time. Jian and Hongjian (2010) [19], in the analysis of the value of saving travel time and the choice of transportation modes, pointed out that travel time is an important and significant point of reference for passengers when choosing a mode of transportation, and that when the distance between cities is less than 720 kilometers, passengers can save more time by choosing HSR over air travel.

Economies of scale refers to the fact that as the scale of production expands, the production cost of an enterprise gradually decreases, which brings about an increase in the economic efficiency of the enterprise. Extending the theory of economies of scale to the transportation industry, the theory of economies of scale in the transportation industry is formed, which means that economies of scale promote cost savings, which will promote the profits of enterprises along the transportation line. By comparing the economies of scale of HSR and traditional railroads, Zhengze and Qingyun (2013) [20] pointed out that the economies of scale of HSR are more obvious than those of traditional railroads, and that compared with ordinary railroads, HSR not only improves the speed, but also improves the range of choices of passengers’ travel time and fare, making the advantages of HSRs more obvious, and the economies of scale more prominent. According to Yu et al. (2017) [21], HSR’s space-time compression effect may greatly reduce the information asymmetry brought about by different geographic locations. Hongchang et al. (2017) [22] measured the economic efficiency of China’s railroad transportation industry by using the improved CCR-DEA model, and found that the efficiency of China’s railroad transportation industry showed a slow upward trend, pointing out that the influencing factors included technological progress, external investment, and changes in industrial structure. At present, China’s transportation infrastructure is gradually improving, but there are still many obstacles in forming economies of scale. Following theoretical analysis of the transportation industry, Haidong (2017) [23] pointed out that China’s transportation market is relatively decentralized in its operation, including a variety of service-oriented small enterprises, and the various links and enterprises in transportation have not reached an effective level of management and unification; this creates the need to integrate transportation resources, and strengthen transportation infrastructure and networking construction, so as to achieve economies of scale. At present, the supply-side reform of China’s road transport market has entered a period of attack, and only by improving the service level and network construction of transportation facilities can we effectively improve the economies of scale of the transportation industry and reduce the operating costs of enterprises. Keqiang (2020) [24] emphasized the need of creating a fair and innovative scale economy in the transportation sector, integrating the available resources into the local environment, beginning with supply-side reform of the roads.

(3) HSR and economic growth: There are two opposing ideas in the academic community on whether HSR can accelerate economic development. According to studies by Ahlfeldt and Feddersen (2018) [25], Yanmei and Yingming (2016) [26], Jun (2017) [27], and others, the process of regional integration and expansion will be facilitated by HSR’s introduction. An objection is that the construction of HSR would surely exacerbate local injustices and jeopardize the growth of underdeveloped areas. For example, Kezhong and Dongjie (2016) [28] verified that the syphoning impact of HSR increased the distance between areas. The main manifestations of this impact were a slower pace of economic development in the city’s peripheral areas and the concentration of economic factors in the core area. According to Yingen and Pengfei (2020) [29], HSR has a two-sided nature for the economic growth of marginal areas, the positive and negative impacts exist at the same time. HSR promotes the economic development of those areas that are rich in specific factors of production of labor and capital, and some areas that have a very weak economic foundation may not be able to get the economic benefits brought by HSR.

(4) HSR and enterprise transformation and upgrading: Using the data of urban enterprises from 1999 to 2011, Mengting and Feng (2018) analyzed the impact of the introduction of HSR on enterprise productivity, and found that HSR has different impacts on enterprises in central cities and peripheral enterprises, and the introduction of HSR suppressed the productivity of enterprises in peripheral cities, with a negative effect of 12.46%; they also analyzed the impact of HSR on the productivity of enterprises and the intrinsic mechanism of the impact of HSR on enterprise productivity. Yihong et al. (2019) [30] use the data about China’s HSR, enterprises, and cities from 2000 to 2011 to construct a double difference model to explore the impact of HSR on enterprises’ exports, and the results show that the exports of enterprises in cities where HSR was introduced increased by 12.7%. Chao and Han (2021) used the data of manufacturing enterprises in Liaoning Province and took the opening of Qin-Shen dedicated passenger line as a quasi-natural experiment to analyze the impact of the introduction of HSR on the vertical division of labor of enterprises through the triple difference method; the results showed that the vertical specialization division of labor of the enterprises whose main means of transportation was via railroads improved by 3–4%, which shows that the introduction of HSR is conducive to the upgrading of the industrial structure of the enterprises along the line, and promotes the improvement of their efficiency and economic benefits.

(5) HSR and science and technology innovation: There are two main explanations of the effect of HSR on innovation in existing studies. One is that HSR promotes the mobility of talents to enhance regional accessibility affecting innovation, and the other is that the introduction of HSR promotes economic agglomeration affecting urban innovation. Based on the 2008–2014 National Innovation Survey enterprise data, Wenhao and Jie (2020) found that the HSR network promotes the movement of highly educated talents, which in turn promotes the scientific and technological innovation capacity of enterprises along the HSR line. Using data from 1,599 listed companies from 2006–2016, Ji and Yang (2020) similarly find that the introduction of HSR promotes the movement of employees with bachelor’s degrees and above, thus enhancing the proportion of human capital and promoting the innovation capacity of enterprises. Yuanchao and Lihua (2019) use panel data of 287 prefecture-level cities in China from 2004–2015 to empirically analyze the impact of the introduction of HSR on regional innovation and regional innovation gaps, and the results show that HSR promotes the mobility of scientific and technological personnel and other innovation factors, which makes HSR have a significant positive impact on regional innovation; but this impact is heterogeneous among different regions, widening the innovation level gap between regions. Xiaoqin and Lei (2006) and Changqi and Yongzhang (2017) argue that economic agglomeration promotes corporate innovation; Ye (2019) analyzes that the introduction of HSR promotes the spatial agglomeration of high-end service industries, which provides the necessary conditions for technological innovation; Dezhu and Shuang (2020) used the panel data of 284 prefecture-level cities in China from 2005 to 2015, and used the introduction of HSRs as a quasi-natural experiment to verify the effect of HSRs on urban innovation at different distance ranges from the central city, finding that the farther away from the central city, the more the impact of HSRs on innovation decreases.

### 2.3 Impact of HSR on the urban-rural income gap

Few studies have been conducted on how HSR affects the income gap between metropolitan and rural regions, and the results have been uneven. Using data from prefecture-level city panel studies and HSR stop frequency data, Fenglong et al. (2018) [31] investigated this kind of effect. Their research indicates that HSR often helps close the wealth difference between urban and rural areas. From the perspective of labor transfer, Yongze and Yan (2019) [32] assessed the effect of the introduction of HSR on the income gap between urban and suburban areas. They achieved this by developing the DID strategy. According to the study’s findings, HSR helped to move employees, therefore lowering the wage gap between urban and rural districts.

**Fig 1:**
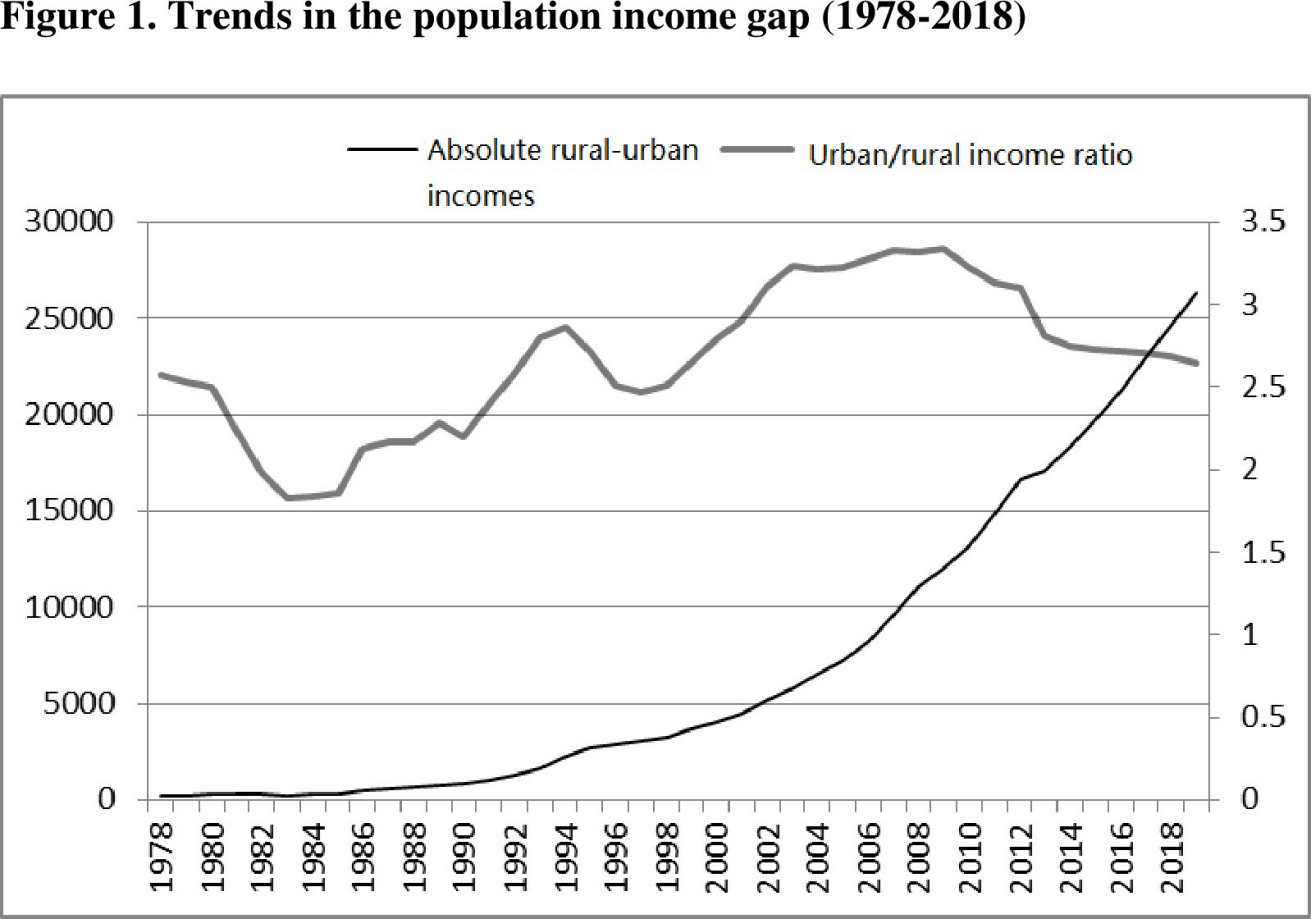
Trends in the population income gap (1978–2018).

Employing panel data from 333 prefecture-level cities and HSR data from China, Feng et al. (2020) [33] conducted an experimental study of the impact of HSR on the urban-rural income gap. They also examined the distance between the main cities of rural areas and HSR terminals. They found that, in general, HSR had little impact on bridging the income gap between urban and rural communities. According to a study by Xinshuo and Suping (2021) [34], the economic effects of HSR on various cities varied. The researchers looked at how it influenced wealth inequality between urban and rural regions at diverse scales of cities. The income gap between urban and rural parts of the country has significantly expanded as a result of the high-speed train system. The Yangtze River Economic Belt’s 108 prefecture-level cities were the subject of Qiang’s research in 2021 [35]; the results (Fig 1) showed that HSR often widened the gap between urban and rural income levels.

### 2.4 Current research status

With regard to research on the urban-rural income gap, scholars at home and abroad have explored the causes of the gap in terms of both endogenous and exogenous factors. In terms of exogenous factors, the urban-rural income gap has widened due to the government’s ongoing urban-biased policies, and in terms of endogenous factors, the factors leading to the existence of the urban-rural income gap mainly include urbanization, the household registration system, labor mobility, financial and educational expenditure, etc. Although scholars have begun to pay attention to the impact of transport infrastructure on the regional gap and the urban-rural gap, the literature on the study of HSRs is very scarce.

Scholars at home and abroad have made positive progress on the economic impact of HSR. Foreign scholars focus on the motivation of HSR development, arguing that it is not due to the economic benefits of HSR, but due to the existence of the positive externality of HSR that the government increases the construction of HSR; on the other hand, domestic scholars’ research on HSR as a transport infrastructure focuses on the impacts of HSR on the economic growth of the region, scientific and technological innovation, the productivity of enterprises, and so on. However, there is relatively little research on the reduction of the urban-rural income gap due to the introduction of HSR. In terms of depth, most of the existing research studies only focus on the measurement of the causal relationship between the two, and there is a lack of in-depth research on the theoretical mechanism of the economic impact of HSR. In this paper, I argue that most of the studies on HSR only focus on one area, and in future, the economic effects of HSR can be sorted systematically and researched based on a global perspective. At the same time, the accessibility of HSR, which impacts regional development, can be deepened, and future studies can further subdivide and decompose HSR accessibility studies.

## 3 Research design

### 3.1 Data source and sample selection

Urban panel data from 285 Chinese prefecture-level cities that were gathered between 2004 and 2018 make up the sample for this study.

(1) Prefecture-level city panel data: The China Statistical Yearbook for Regional Economy, the China City Statistical Yearbook, and the statistical yearbooks of the several provinces and municipalities all contributed to the compilation of the vast bulk of the data. Only a minor quantity of data was procured from the websites of the statistics offices of the several provinces and municipalities. In order to guarantee that the information given was correct and exhaustive, the author of this piece chose to base the data range on the whole of the city rather than on the specific municipal districts. The author of this study made the decision not to include Sansha City, Danzhou City, Xiangxi Prefecture, or any of the other prefecture-level cities found within the Tibet Autonomous Region that are situated outside of Lhasa City. Nevertheless, Bijie City and Tongren City are included, even though their administrative divisions shifted while the research was being carried out.

(2) Details on the commencement of HSR routes: The China Railway Yearbook, news and announcements posted on the China Railway Corporation 12306 website, and Qunar are sources of information on the planning and inauguration dates for HSR lines as well as the dates for the HSR networks becoming operational in each city.

### 3.2 Variable definitions and model construction

#### 3.2.1 Definitions of variables

(1) Dependent variables: The imbalance in revenue amongst urban and rural regions is the explanatory variable in this particular research. The Gini coefficient, the difference in income levels among both urban and rural areas, and the ratio of urban to rural income are the three main factors used to calculate the urban-rural income gap. Drawing on Qinlin et al. (2023) [36] and Beibei et al. (2022) [37], in this research I compare the per capita net earnings of rural inhabitants to the per capita disposable income of urban residents in each prefecture-level city to measure the income gap between urban and rural residents. The objective of the comparison is to ascertain the extent to which urban inhabitants earn more than those who dwell in rural regions.

(2) Independent variables: Referring to Xinshuo and Suping (2021) [6] and Yana (2022) [38], the determination to use HSR as a policy is picked as the primary variable for the purpose of explanation in this paper. The aim of this essay is to provide more insight into this choice. This coverage extends to the years from 2004 to 2018, both inclusive. If a city on the level of a prefecture has an HSR station, then that city is regarded as having HSR. This is the most important factor that was considered when deciding whether or not there are HSRs in an area. Numerous researchers are of the opinion that the policy dummy variable, which is produced by the interaction term of the city dummy variable (id) and the year dummy variable (year) of the HSR, has the potential to act as a proxy variable for the introduction of an HSR. This hypothesis was arrived at by taking into account the fact that the policy dummy variable was generated. If it opens before July in a given year, it counts as the start of the current year; if it starts after July of a given year, the actual opening time of the HSR counts as the start of the following year. The data pertaining to the HSR is simultaneously treated as follows: if it opens before July of a given year, it counts as the start of the current year; if it starts after July of a given year, it counts as the start of the following year. If it opens before to July of the current year, then it is considered to have begun in the previous year.

(3) Control variables: There are many influencing factors that cause the income gap between urban and rural residents to widen or narrow, in addition to the focus of this paper–whether the prefecture-level cities have HSRs or not. With reference to the studies of Mingwang and Jiaping (2019) [39], Muchen et al. (2020) [40], Beibei et al. (2023) [41] and He and Qinxian (2018) [42], I selected Level of economic development of prefecture-level cities (pgdp), degree of openness to the outside world (Open), Industrial structure (tery), Scale of financial development (fde), Level of science and technology development (tec), Level of educational development (edu), Wage level (lnwage), and Unemployment rate (une) as control variables.

Level of economic development of prefecture-level cities: Since economic growth has a substantial influence on citizens’ income, in this study I calculate the logarithm of the Gross Domestic Product (GDP) of prefecture-level cities.

By examining a nation’s level of economic openness to the rest of the world, one may determine how open its economy is to the rest of the world. International trade has an immediate effect on how many people are employed in the economy. The proportion of each city prefecture’s GDP that is supplied by all imports and exports from outside is calculated in this study. The US dollar conversion units for total imports and exports are calculated using the average exchange rate between the US dollar and the Chinese yuan that can be accessed on the website Exchange Rate Check.

Industrial structure: The proportion of tertiary sector production in GDP is used to represent the industrial base.

Scale of financial development: Agriculture and rural development can receive financial help through financial development schemes. However, as society and the economy evolve, financial attention is increasingly directed toward metropolitan areas, which has led to a fast expansion of urban economies. Due to a lack of financial services in rural areas, urban and rural communities have different financial circumstances. The level of financial development also affects the growth of rural agriculture and, in turn, the income of farmers. In this research I gauge the level of financial development based on the ratio of financial institutions’ end-of-year RMB loan balances to domestic production.

Level of science and technology development: The level of economic development may be gauged by the pace of scientific and technological advancement, which is also a major element in the increase in per capita income. The degree of science and technology in prefecture-level municipalities is shown by the ratio of research and development investment to fiscal expenditure.

Education expenditure: Since 2007, China’s government has been allocating an ever larger part of its total expenditure to education. The amount that is spent on education has led to an improvement in both the quantity and the quality of the education that is received by the population, which has in turn led to an increase in the population’s ability to participate in the social division of labor. The logarithm of the total amount spent on education is the measurement that is used for this statistic.

Wage level: Since residents’ earnings are a good indicator of their quality of life, in this research I calculate the level of residents’ salaries using the logarithm of the average pay for urban workers.

Unemployment rate: The unemployment rate is a key factor in determining the average income of city inhabitants and provides insight into the employment situation of the urban labor force. The ratio of officially recorded urban unemployment to the entire population is used to calculate the unemployment rate.

Table 1 below summarizes all the variables:

**Table 1:** Definition of variables.

Variable Type	Name	Symbolic	Definition
Explained variables	Income gap between urban and rural residents	Gap	Per capita disposable income of urban residents/per capita disposable income of rural residents
	Per capita income of urban residents	Uincome	Urban per capita disposable income in logarithms
	Net income of farmers	Rincome	Disposable income per rural inhabitant in logarithms
Explanatory variables	Introduction of HSR	Hsr	Assign a value of 1 to the introduction of HSR, and 0 to non-introduction
Control variables	Level of economic development	pgdp	Per capita GDP taken as logarithm
	Level of openness to the outside world	Open	Total import/export trade/GDP
	Industrial structure	tery	Tertiary sector output/GDP
	Scale of financial development	fde	Total Renminbi loans/GDP
	Level of scientific and technological development	tec	Science and technology expenditure/fiscal expenditure
	Level of educational development	edu	Education expenditure taken as log
	Wage levels	lnwage	Average wage of urban workers taken as a logarithm
	Unemployment rate	une	Unemployed persons/total population at the end of the year

#### 3.2.2 Model construction

In this research I approach the inauguration of the HSR as a form of quasi-natural experiment and use the DID method to investigate and confirm the effect of the introduction of the HSR on the discrepancy in per capita disposable income between urban and rural populations.

Since, theoretically, the building of an HSR would have an impact on a city’s economic status, employment, and a number of other economic indicators, the decision to introduce HSR or not will cause a difference in the city’s economic level. In this paper I split the sample cities into experimental and control groups based on whether or not an HSR was officially launched in each, allowing us to compare the effect using DID. In this paper I use the multi-temporal double-difference as well as additional controls for time and city personal effects to evaluate the impact of the introduction of HSR since the traditional double-difference may not be applicable because the policy is not a one-time event but rather occurs annually in each city. The model can be seen below.

$${GAP}_{it}=\beta_{0}+\beta {HSR}_{it}+\alpha X_{it}+\gamma_{i}+\delta_{t}+\varepsilon_{it}$$ (1)

where *GAP*_*it*_ reflects the wealth discrepancy between cities and suburban societies, as calculated by the ratio of per capita disposable income of urban inhabitants to per capita net income of countryside residents. i is for the ith city, while t stands for the tth year. The constant term is denoted by *β*_0_. *HSR*_*it*_ indicates whether or not a city is accessible by HSR. *β* is the *HSR*_*it*_ coefficient, which is used to gauge how the introduction of HSR has affected the urban-rural wealth disparity. *HSR*_*it*_ = 1 indicates that the ith city launches HSR in year t, and if the coefficient is positive, it means that the introduction of HSR widens the income gap between urban and rural residents. Numerous control variables make up *X*_*it*_. *α* denotes whether the control variables have a favourable or unfavourable effect on the dependent variable, the difference in earnings between cities and suburbs. The letters *δ*_*t*_, *γ*_*i*_, and *ε*_*it*_ stand for the temporal fixed effect, the urban fixed effect, and the random error term, respectively.

In this paper I adopt the following framework to evaluate the influence of HSR operation on the disposable incomes of urban and rural inhabitants, respectively. To analyze how the introduction of HSR may affect the income disparity between urban and rural populations, this model is required. Here is a process for developing a two-way fixed effects model.

$$ln{urbanincome}_{it}=B_{0}+\beta {HSR}_{it}+\alpha X_{it}+\gamma_{i}+\delta_{t}+\varepsilon_{it}$$ (2)

$$ln{ruralincome}_{it}=B_{0}+\beta {HSR}_{it}+\alpha X_{it}+\gamma_{i}+\delta_{t}+\varepsilon_{it}$$ (3)

where ln*urbanincome*_*it*_ and ln*ruralincome*_*it*_ represent the logarithms of urban residents’ disposable income per capita and rural residents’ disposable income per capita, respectively, and where the variables in the model are consistent with the above model.

## 4 Empirical results and analysis

### 4.1 Descriptive statistical analysis

Table 2 below demonstrates the results of the descriptive statistics:

**Table 2:** Descriptive statistics results.

Variable	Obs	Mean	S.D.	Min	Max
gap	4275	2.588	0.624	1.296	6.885
hsr	4275	0.253	0.434	0	1
pgdp	4275	10.211	0.791	4.595	12.579
open	4275	0.181	0.325	0.085	4.622
trey	4275	0.378	0.09	0.086	0.853
fde	4275	0.838	0.537	0.075	7.45
tec	4275	0.012	0.131	0.01	0.207
edu	4275	12.408	1.009	6.855	15.298
wage	4275	10.391	0.565	8.509	12.678
unemploy	4275	0.006	0.005	0.004	0.115

### 4.2 Correlation analysis

Correlation tests are run on each variable in this stage to check for the possibility of spurious regression brought on by various collinearities. The table below (Table 3) shows that there is no proof of multiple collinearities between the variables. VIF tests were performed on each variable in order to better investigate the many collinearities. The fact that the mean is 2.14 and the highest VIF is 4.18—both of which are less than 10—indicates that numerous collinearities have minimal impact.

**Table 3:** Correlation analysis.

	gap	pgdp	open	tery	fde	tec	edu	wage	une
gap	1								
pdp	-0.514	1							
open	-0.205	0.303	1						
tery	-0.053	0.256	0.177	1					
fde	-0.064	0.298	0.154	0.406	1				
tec	0.311	0.549	0.317	0.269	0.243	1			
edu	-0.226	0.601	0.101	0.305	0.266	0.455	1		
wage	-0.297	0.778	0.073	0.290	0.359	0.431	0.747	1	
une	-0.193	0.240	0.144	0.092	0.198	0.092	-0.111	0.046	1

### 4.3 Parallel trend test

**Fig 2:**
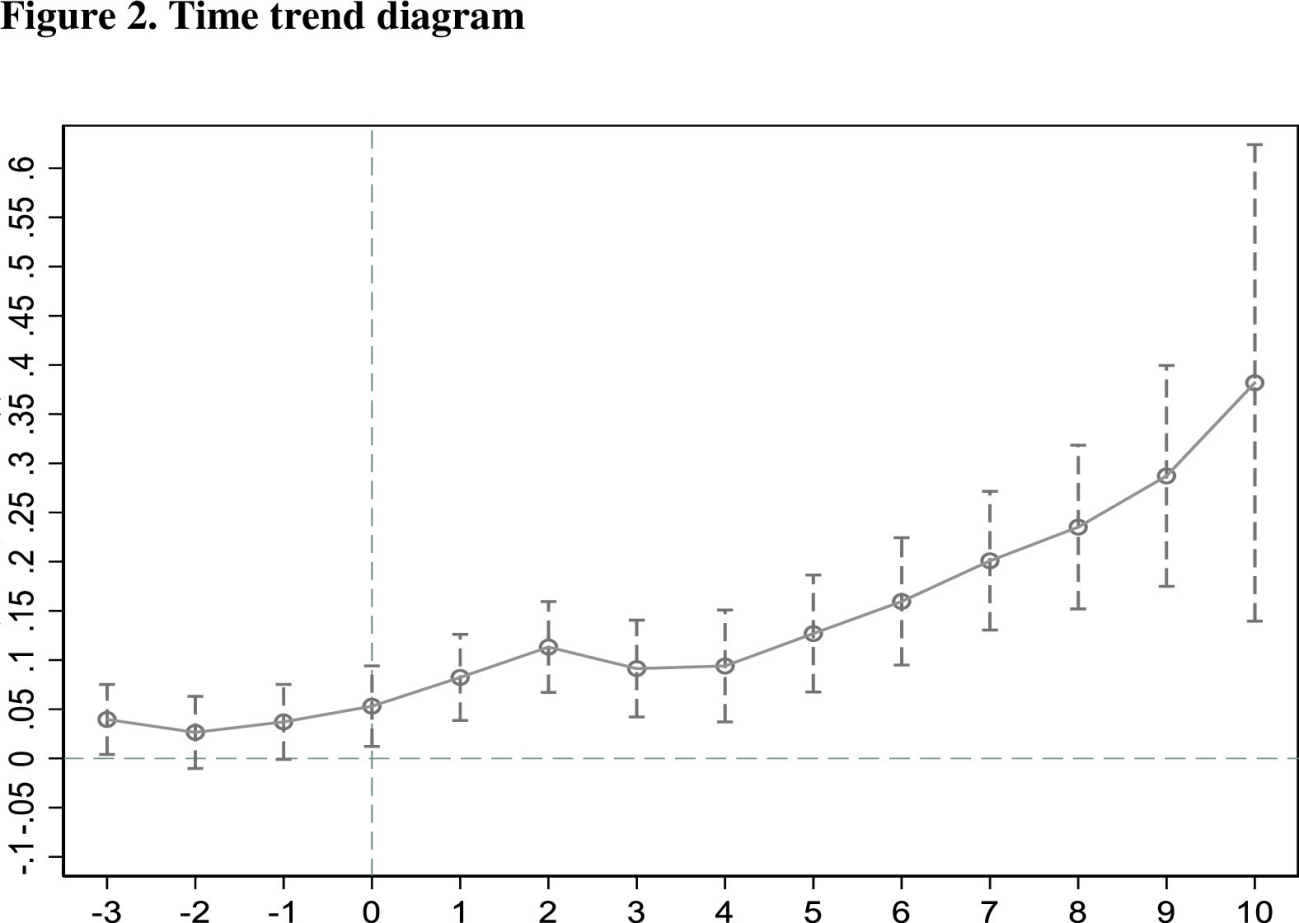
Time trend diagram.

The explanatory variables in the two regions must have the same development trend and be similar before the policy’s implementation in order to satisfy the parallel trend test, which is the cornerstone of DID. The income disparity between urban and rural residents would have been changing before the HSR’s construction in a similar manner as the equivalent control group. On this premise, it may be possible to determine how policy will affect the two samples when the HSR is launched. Finally, the authenticity and effectiveness of all chosen data and models are verified by constructing parallel trend graphs for parallel trend testing. The data in this research span a 15-year period from 2004 to 2018. The year 2008 was when HSR was initially implemented, hence in this study I used that year as the starting point for event analysis. For the effect of the policy to be deemed acceptable, the parallel trend test must be successful. This is the parallel trends chart (Fig 2):

The abscissa of the previous graph serves as a representation of the time period, with 0 points designating the start of the policy in 2008. The ordinate indicates the estimated treatment effect coefficient, and 0 points reflect an estimated coefficient value of 0. According to the outcomes of the parallel trend test, which are shown in the above figure, the projected value of the treatment impact coefficient fluctuated by about 0 before the policy was implemented. The wealth disparity between urban and rural populations was negligible previous to the high-speed train’s building projects. After the policy was put in place, the expected value began to vary from 0 points. The positive coefficient proved that the experimental group benefited from the implementation of the policy. Given that there was initially not much of a difference in the income gap between the two sample groups, it is indisputable that the financial difference between urban and rural populations was negatively impacted by the expansion of HSR. The wealth difference between urban and rural populations is changing over time as a result of HSR. The policy did not have any significant effects for the first four years after it was implemented, but beyond that point it started to become apparent, indicating a trend of increase year after year. The effect of HSR on the disparity in wealth between urban and rural populations may be observed in phases. (1) In the three years prior to the introduction of HSR in each city, the estimated value of the treatment effect coefficient was non-significant and close to 0, supporting the parallel trend theory and demonstrating that both the experimental and control groups had the same trend. (2) In the fourth year after its introduction, it became evident that the HSR exerted a favorable influence on the income gap, and the treatment effect grew with time.

In conclusion, the analysis shown in the aforementioned figure demonstrates, on the one hand, that the DID technique used in this work passes the parallel trend test and accurately assesses how the inauguration of HSR would affect revenue inequality among both urban areas and rural places; on the other hand, the study’s findings support the lagging aspects of the introduction of HSR strategy that have an effect on the disparity in income between urban and rural regions. The income disparity between urban and rural regions was originally scarcely affected by the strategy, but by the fourth year, it had begun to widen rapidly. HSR is still in its infancy and, due to its higher cost, has not yet had a substantial impact on the general population as much as other types of transportation, including ordinary trains, and due to its limited availability across the country. A growing number of individuals are choosing to utilize HSR networks as they proliferate throughout the nation. HSR, which allows for labor mobility, has a gradual economic impact.

### 4.4 Benchmark regression analysis

Prior to doing the regression analysis, a Hausman test was run on the model, and a p value of 0 suggested that a fixed-effect model should be looked at. This section employs a two-way fixed-effects model for multi-period DID. The following table (Table 4) shows the regression results for the incremental inclusion of the control variables in columns (1), (2), (3), (4), (5), and (6) and the benchmark regression result in columns (1), (2), (3), (4), (5), and (6). While column (8) displays the result after taking into account all control factors, column (1) displays the regression results without any control variables. HSR is referred to as Hsr, and a collection of control variables is referred to as pgdp-edu.

**Table 4:** Regression analysis.

	(1)	(2)	(3)	(4)	(5)	(6)
Hsr	0.0722***	0.0568***	0.0569***	0.0599***	0.0656***	0.0653***
	[0.013]	[0.013]	[0.013]	[0.013]	[0.013]	[0.013]
Pgdp		-0.289***	-0.285***	-0.276***	-0.274***	-0.275***
		[0.054]	[0.055]	[0.056]	[0.062]	[0.062]
Open			-0.0197	-0.0292	-0.0414	-0.039
			[0.042]	[0.042]	[0.041]	[0.041]
Trey			0.0592	0.0812	0.0879	0.086
			[0.079]	[0.081]	[0.081]	[0.081]
Fde				0.024	0.0275	0.0282
				[0.016]	[0.016]	[0.016]
Tec				-0.637	-0.568	-0.542
				[0.437]	[0.425]	[0.424]
Edu					-0.106***	-0.106***
					[0.031]	[0.031]
Wage					0.163***	0.163***
					[0.038]	[0.038]
Unemp						-1.743
						[0.950]
_cons	2.570***	5.529***	5.469***	5.357***	4.953***	4.957***
	[0.005]	[0.555]	[0.566]	[0.577]	[0.520]	[0.519]
Individual effect	Control	Control	Control	Control	Control	Control
Time effect	Control	Control	Control	Control	Control	Control
N	4275	4275	4275	4275	4275	4275
adj.R-sq	0.844	0.85	0.85	0.85	0.852	0.852

Standard errors in brackets

* p<0.05,

** p<0.01,

*** p<0.001

The coefficient of HSR installation in the model without any control variables is 0.0722, and at the 1% level, it is statistically meaningful. This can be seen in the graph above. The disparity in wealth between city dwellers and country folk has decreased as a consequence of the advent of HSR. The coefficient of the introduction of the HSR remained continuously positive and mostly steady when the control variable was progressively introduced from (2) to (5), and all tests passed the 1% significance threshold. This demonstrates that the implementation of HSR has the effect of growing the gap between the earnings of urban and rural communities. The regression results for each model covariate are shown in the next column (6). The statistically significant and positive HSR activation coefficient at the 1% level is 0.0653, which is high. This illustrates that the wealth divide across inhabitants of urban and rural regions has substantially risen as a result of the construction of HSR.

The economic development level coefficient was significantly negative compared to the control variables at -0.275 at the 1% level. This demonstrates how greater economic development has a beneficial impact on the revenue difference between metropolitan and rural communities. In direct proportion to the city’s level of economic development, both the number of individuals who will profit from economic growth and the rise in the average income of city inhabitants will climb. Committed to economic development and increasing the absolute amount of the economy generally continues to be the most effective means for speeding up the growth in people’s incomes. According to the coefficient of openness to the outside world, which is -0.039, the income disparity among urban districts and countryside districts decreases as access to the outside world increases. The coefficient for the industrial structure is 0.086, indicating that when the industrial structure is improved, the wealth inequality between urban and rural residents expands. The tertiary industry’s share of the GDP is used to assess this disparity. The advantages of improving the industrial structure for urban residents are greater than those for rural people since this development has increased the number of jobs and employment opportunities available to urban citizens. If we wish to close the income gap, we must better integrate the workforce in rural areas. According to the scale’s positive impact coefficient on the income difference between parts of the city and the countryside, there is a positive correlation between the financial development scale and the increasing income disparity. As more money comes into the city and resources are concentrated there, urban growth has taken priority over rural development in recent years. This significantly affects how urban inhabitants’ incomes rise. The wage inequality between metropolitan and rural regions has grown as a result of an increase in the financial development index. The coefficient of science and technology development level of -0.542, which does not go above the 10% significance threshold, demonstrates that the revenue disparity within rural and urban regions has decreased. Science and technology advancements have made it easier for low-skilled people in rural areas to embrace new technologies and become more productive workers, which has boosted the economy of rural communities. At the 1% level, the coefficient of education spending is clearly negative. As education investment increases, the economic divide between urban and rural people decreases. As a result, the government has to spend more money on education. Since 2007, China has increased its educational expenditure, particularly on state-implemented free compulsory education in rural areas and comprehensive training for the idle rural labor force, enhancing the skills and income of farmers. The urban wage coefficient is 0.163%, which is statistically significant at the 1% level. This illustrates how when wages for urban workers grow, the disparity of urban and rural populations’ incomes expands. The recent influx of a sizable number of migrant workers looking for employment in urban areas has provided a considerable source of income for individuals residing in rural areas. Often, migrant workers make less money than their colleagues in urban areas. Emerging imbalance in wealth across urban and rural locations is mostly caused by the rising wage revenue disparity. The coefficient for the unemployment rate is -1.743%. Urban unemployment has an effect on the average compensation of urban workers, as shown by the inverse relationship between the wage inequality contrasting city dwellers and country folk and unemployment. Over the last several years, the wealth gap between urban and rural areas has typically decreased, but the introduction of HSR has considerably widened the difference. This is most likely due to the fact that the availability of HSR makes it simpler for individuals to move money and labor between cities, which affects both the earnings of urban and rural residents.

### 4.5 Robustness analysis

#### 4.5.1 Single-phase DID-test

According to the multi-point DID research mentioned above, increased economic inequality between urban and rural populations would occur following the development of an HSR route. However, the day a high-speed train commences operation differs depending on the location. In other words, since the program for building HSRs is implemented year by year, projections may be erroneous. In this section, DID analysis is used once again, and reference is made to Yongze and Yan (2019) [34]. The only samples that will be preserved are those from the 2011 introduction of HSR, as well as all control groups that did not make use of HSRs. Twenty HSR stations were ultimately opened in 2011 as a result of the reorganization, as opposed to the 97 HSR stations that remained closed. Following a regression study, it was determined that the variable coefficient for the start of an HSR was 0.127, which is much higher than the 1% limit. This was discovered after the data were subjected to scrutiny. This is in line with the results of the analysis that was quoted before and demonstrates that the introduction of HSR has dramatically contributed to the widening wealth imbalance that exists between cities and rural areas. Urban and rural inhabitants’ respective absolute incomes are shown in columns (2) and (3), respectively, to show the effect that HSR has had, net-positively, on both these populations (Table 5).

**Table 5:** Regression results of traditional differential method.

	(1)	(2)	(3)
	Gap	Urincome	Ruincome
Hsr	0.127***	0.00763	-0.0257***
	[0.024]	[0.008]	[0.007]
Pgdp	-0.373***	0.0957***	0.184***
	[0.048]	[0.015]	[0.015]
Open	0.0336	0.0114	0.00277
	[0.048]	[0.017]	[0.012]
Trey	0.371**	0.125***	0.0212
	[0.132]	[0.036]	[0.037]
Fde	-0.0320*	0.000946	0.0102
	[0.014]	[0.006]	[0.007]
Tec	1.096	-0.7	-0.426
	[0.813]	[0.388]	[0.275]
Edu	-0.0118	0.0347*	0.0407*
	[0.041]	[0.016]	[0.018]
Wage	0.291**	0.155***	0.0807***
	[0.089]	[0.024]	[0.024]
Unemp	-3.255*	-1.294	-0.223
	[1.576]	[0.704]	[0.407]
_cons	3.444***	6.712***	5.599***
	[0.939]	[0.250]	[0.278]
Individual effect	Control	Control	Control
Time effect	Control	Control	Control
N	1755	1755	1755
adj.R-sq	0.856	0.981	0.987

Standard errors in brackets

* p<0.05,

** p<0.01,

*** p<0.001

#### 4.5.2 Special samples excluding, all explanatory variables lagging by one stage, PSM-DID test

(1) Because provincial cities have an advantage over other cities in terms of economic strength, urban size, population quantity, and quality, and other cities are difficult to compare in these areas, the conclusions may not be accurate. In this section, the sample is modified to exclude a number of provincial and vice-provincial capitals, and the regression analysis is redone to see how reliable the conclusions are. The first column of the table below displays the regression analysis findings. After the specific sample is removed, the coefficient of HSR inauguration, which is positive and significant at the level of 1%, implies that the introduction of HSR tremendously broadened the income disparity that formerly separated urban and rural areas. The validity of the findings of the regression benchmarks discussed before is supported by this discovery.

Based on the above and Hua and Feng (2019) [43], the HSR’s launch has been postponed, taking into account how it would affect the income of urban and rural populations over time. To avoid the simultaneous equations bias, a first-phase lag is applied to all control variables before the regression is repeated. The results of the regression analysis, which are shown in column (2) of the above table (Table 6), reveal that the coefficient of HSR activation is positive and statistically significant at the 1% level, effectively confirming the validity of the empirical test’s conclusions.

**Table 6:** Regression results.

	(1)	(2)	(3)
	Delete special sample	All explanatory variables lag by one stage	PSM-DID
Hsr	0.0632***	0.0701***	0.0616***
	[0.0142]	[0.0132]	[0.0139]
Pgdp	-0.258***	-0.224***	-0.283***
	[0.0633]	[0.0544]	[0.0747]
Open	-0.0021	-0.059	-0.191***
	[0.0422]	[0.0417]	[0.0550]
Trey	0.097	0.189*	0.0194
	[0.0931]	[0.0863]	[0.0861]
Fde	0.0249	0.0372*	0.0286
	[0.0187]	[0.0162]	[0.0209]
Tec	-0.255	-0.0621	0.766
	[0.4457]	[0.4553]	[0.6974]
Edu	-0.0800*	-0.0986**	-0.114**
	[0.0332]	[0.0315]	[0.0381]
Wage	0.175***	0.154***	0.164***
	[0.0387]	[0.0378]	[0.0451]
Unemp	-1.93	-1.484	-1.342
	[1.0306]	[0.9427]	[1.2368]
_cons	4.337***	4.383***	5.181***
	[0.5435]	[0.5213]	[0.5899]
Individual effect	Control	Control	Control
	Control	Control	Control
N	3810	3990	3759
adj.R-sq	0.8558	0.8597	0.8542

Standard errors in brackets

* p<0.05,

** p<0.01,

*** p<0.001

A two-way fixed-effect model may be employed in the research indicated above to control part of the endogenous issue, and a multi-point DID is used to estimate the net effect of the introduction of HSR on the disparity in wealth amongst urban and rural dwellers. However, this method falls short in addressing the endogenous issue brought about by sample selection bias. In this section, the endogeneity problem, as well as the appropriate sampling bias problem, may both be tackled with the use of the PSM-DID methodology. Since there may be intrinsic variations between the economic characteristics of the experimental and control groups, this section employs the near neighbor matching approach to identify appropriate control group samples for the experimental group. This method, which makes use of the observable characteristic variable, seeks to identify cities that do not have HSR but do share as many characteristics as possible with cities that do have HSR. The goal of this method is to reduce the gap between the experimental group and the control group and to make comparisons between the two groups easier. I used the cities that always had access to high-speed trains throughout the sample period as the treatment group. After that, I matched the experimental group year by year using the propensity score matching approach, which is based on the 1:3 near neighbor matching strategy with replacement sampling.

The sample balance is satisfied following a propensity score match and the experimental and control groups do not vary in any way that is noteworthy, as shown by the fact that the p-value of the difference between the means of each control variable in the experimental and control groups does not exceed the 10% significance level after matching. After year-by-year matching, I reapplied double-difference to the matched sample to perform regression. Column 3 of the table above (Table 6) contains a list of the analyses conclusions. In compliance with the coefficient of HSR inauguration, which is still statistically significant and positive at the 1% level, the revenue discrepancy between urban and rural areas grows. The basic pattern of this discovery is in agreement with the regression findings presented earlier.

#### 4.5.3 The placebo tests

I am taking into consideration the likelihood that there might be more unobservable factors impacting on how a high-speed urban railway starts. HSR and the revenue imbalance between metropolitan and rural districts may also have a false regression problem. In addition to the effects of HSR’s initial components, there may be additional unintended and unobservable factors that influence the widening or shrinking of the wealth disparity within cities and remote areas. A placebo test is utilized in this research as additional proof because of this (Table 7). In this section I constructs the dummy variable HSR1 as a control, utilizes the DID for regression analysis, and uses the hypothetical times of the introduction of HSR in the second, third, and fourth years before the actual year when the HSR starts operation in each city. Pingui’s (2019) [44] and other researchers’ studies are cited in order to achieve this. Even if the coefficient of HSR is not substantial, the installation of HSR has a large causal influence on the economic disparity among cities and the surrounding countryside. HSR’s introduction has a sizable causal effect on urban-rural wealth inequality, but it has no appreciable influence on any of the explanatory factors during the virtual era, indicating a lack of the virtual disposition effect suggested in this paper.

**Table 7:** Placebo test results.

	(1)	(2)	(3)
	2007	2006	2005
HSR1	0.0133	0.0102	-0.00511
	[0.016]	[0.016]	[0.018]
Pgdp	-0.286***	-0.285***	-0.286***
	[0.064]	[0.064]	[0.064]
Open	-0.0481	-0.0478	-0.048
	[0.042]	[0.042]	[0.042]
Trey	0.0552	0.0566	0.0514
	[0.080]	[0.081]	[0.080]
Fde	0.0256	0.0256	0.0254
	[0.016]	[0.016]	[0.016]
Tec	-0.264	-0.261	-0.256
	[0.445]	[0.444]	[0.445]
Edu	-0.0981**	-0.0983**	-0.0979**
	[0.031]	[0.031]	[0.031]
Wage	0.156***	0.156***	0.156***
	[0.038]	[0.038]	[0.038]
Unemp	-1.832	-1.827	-1.836*
	[0.935]	[0.935]	[0.934]
_cons	5.078***	5.075***	5.084***
	[0.522]	[0.523]	[0.524]
Individual effect	Control	Control	Control
Time effect	Control	Control	Control
N	4275	4275	4275
adj.R-sq	0.851	0.851	0.851

Standard errors in brackets

* p<0.05,

** p<0.01,

*** p<0.001

## 5 Heterogeneity analysis

### 5.1 Regional analysis

To test and explore the influence of HSR’s introduction on different places, cities throughout the country are separated into east, central, and western segments. The table above (Table 8) shows that since the HSR’s introduction, urban-rural wealth inequality has grown in the eastern and western regions while decreasing in the central area. First, the coefficient of the main explanatory factor, based on (1), is 0.0702, which is substantially greater than 1%. The HSR policy’s openness greatly reduces the economic disparity among urban and rural communities in eastern China, which is reflected in a widening income gap between the two. The coefficients of level of economic development and level of science and technology are both negative and statistically significant at the 1% level, showing that the income gap between urban and rural regions has shrunk as a consequence of the progress of both. The income gap between eastern urban and rural areas has shrunk as a result of rising education expenditure and a high unemployment rate. There is a very strong negative association between education spending and the unemployment rate at the 5% level. The HSR coefficient, which is given by equation (2) to be -0.0471, is statistically significant at the 10% level. HSR has a reversing effect on the economic difference among urban and rural populations in the central region, narrowing the divide between them. The opening coefficient for the HSR is 0.165, which is statistically significant at the 1% level, as indicated in column 3. The income disparity between urban and rural residents has narrowed since HSR arrived in this area. This is because the HSR was introduced later in the area’s western part, which had slower economic development than the area’s eastern part. Local resources like money and labor could relocate to the more developed eastern and central regions if transportation infrastructure continues to be enhanced.

**Table 8:** Regional regression results.

	(1)	(2)	(3)
	The eastern region	The central region	The western region
Hsr	0.0702***	-0.0471*	0.165***
	[0.016]	[0.023]	[0.033]
Pgdp	-0.172***	-0.304***	-0.222*
	[0.043]	[0.070]	[0.111]
Open	-0.0447	0.278*	-0.0826
	[0.043]	[0.118]	[0.092]
Trey	-0.106	-0.0583	0.374*
	[0.116]	[0.150]	[0.159]
Fde	0.0159	0.0271	0.104*
	[0.023]	[0.021]	[0.042]
Tec	-2.024***	-0.239	3.646*
	[0.475]	[0.692]	[1.808]
Edu	-0.120**	-0.031	0.00637
	[0.040]	[0.073]	[0.060]
Wage	0.0583	0.268**	0.164**
	[0.066]	[0.083]	[0.063]
Unemp	-3.688**	6.958*	6.283*
	[1.355]	[2.872]	[2.697]
_cons	5.104***	3.233**	3.129**
	[0.723]	[1.103]	[1.192]
Individual effect	Control	Control	Control
Time effect	Control	Control	Control
N	1980	1095	1200
adj.R-sq	0.773	0.849	0.837

Standard errors in brackets

* p<0.05,

** p<0.01,

*** p<0.001

### 5.2 City level analysis

The introduction of HSR is illustrative of the variety of urban regions because of the uneven distribution of HSR stations that exist in the different districts. Following the modification of the urban scale division standard by the State Council, the sample of 285 prefecture-level cities was divided into five levels according to the urban permanent resident population standard. This marked the beginning of the heterogeneity study of the cities at each level. The columns labeled (1), (2), (3), (4), (5), respectively display the cities that fall into the first, second, third, fourth, and fifth categories of urban density (Table 9). In light of the results, the construction of HSR might have a number of different impacts on cities of different sizes. HSR was shown to reduce the urban-rural income gap between the first and third tiers of cities, but to increase it between the second, fourth, and fifth tiers. For fourth-tier cities, the importance threshold was 10%, while for fifth-tier cities, it was 1%. First-tier cities are also considerably superior to other hierarchical cities in terms of the full range of advantages and governmental regulations related to urban size and economic growth level. The discrepancy in wealth between urban and rural locations has narrowed with the introduction of HSR in major cities. The speed of urban economic development is faster than that of rural regions due to the limited economic strength of cities in the fourth and fifth tiers, leading to a rising income disparity within urban and rural regions.

**Table 9:** Regression of subdivided samples by city level.

	(1)	(2)	(3)	(4)	(5)
	First tier	Second tier	Third tier	Fourth tier	Fifth tier
Hsr	-0.0654	0.0399	-0.0169	0.0438*	0.167***
	[0.057]	[0.029]	[0.019]	[0.022]	[0.032]
Pgdp	-0.0938	-0.116	-0.319***	-0.476***	-0.191*
	[0.079]	[0.089]	[0.066]	[0.056]	[0.093]
Open	-0.0279	0.0651	-0.206	-0.199*	-0.251***
	[0.051]	[0.052]	[0.122]	[0.100]	[0.073]
Trey	0.898	0.333*	-0.328*	-0.184	0.538**
	[0.580]	[0.143]	[0.143]	[0.125]	[0.174]
Fde	0.269**	0.0198	0.0632	-0.0146	0.118
	[0.084]	[0.040]	[0.048]	[0.015]	[0.061]
Tec	-1.396	-4.718***	0.0586	-0.247	-0.675
	[1.185]	[1.311]	[0.650]	[1.584]	[0.923]
Edu	-0.447***	-0.364***	0.0783	-0.0963	-0.124*
	[0.095]	[0.090]	[0.097]	[0.052]	[0.052]
Wage	0.481**	0.269	0.277**	0.281**	0.0816
	[0.172]	[0.138]	[0.104]	[0.092]	[0.053]
Unemp	-14.29***	28.39**	-1.792	-0.869	-2.661
	[3.361]	[8.785]	[1.081]	[1.629]	[1.776]
_cons	3.625	5.260**	2.009	5.832***	5.016***
	[2.145]	[1.618]	[1.184]	[0.966]	[1.044]
Individual effect	Control	Control	Control	Control	Control
Time effect	Control	Control	Control	Control	Control
N	210	450	1050	1260	1305
adj.R-sq	0.773	0.844	0.829	0.859	0.857

Standard errors in brackets

* p<0.05,

** p<0.01,

*** p<0.001

## 6 Research on mechanisms

### 6.1 Decrease in the income of the rural population

It is necessary to differentiate the impacts on the earnings of urban and rural residents when analyzing the increasing influence of HSR construction on the urban-rural wealth disparity. In this section I investigate how HSR’s introduction affects urban and rural inhabitants differently, using Model 2 from the previous section and independently regressing the earnings of urban and rural dwellers. Income logarithms are used to compare the incomes of urban and rural residents.

The columns (1) and (2) of Table 10, respectively, indicate the effects of HSR on rural and urban residents’ income. Columns (5) and (6) show the regression results after excluding cities in the Yangtze River Delta, Pearl River Delta, Beijing, Tianjin, and Hebei major economic zones, while columns (3) and (4) show the regression results after excluding provincial and vice-provincial capitals from the entire sample. While none of the three examples had a substantial effect on urban residents, the introduction of HSR had a considerably negative influence on rural residents’ income. This shows that the declining net income per capita of rural residents is the cause of the widening wealth divide between urban and rural regions. The influence of HSR on rural economies is substantially less than on urban economies due to the distance between HSR stations and rural areas, as well as the minor impact that their introduction will have on the lives of rural populations.

**Table 10:** Regression results of the introduction of HSR on the income of urban and rural residents.

Project	Full sample		Excluding large cities		Removal of the three economic spheres	
	(1)	(2)	(3)	(4)	(5)	(6)
HSR	-0.0193***	0.00138	-0.0204***	-0.00252	-0.0123*	0.000249
	[0.004]	[0.004]	[0.004]	[0.004]	[0.005]	[0.005]
Control variables	control	control	control	control	control	control
_cons	6.452***	7.593***	6.614***	7.497***	6.001***	7.683***
	[0.301]	[0.210]	[0.306]	[0.224]	[0.275]	[0.219]
Individual Effect	control	control	control	control	control	control
Time effect	control	control	control	control	control	control
N	4275	4275	3810	3810	3240	3240
adj.R-sq	0.986	0.982	0.986	0.981	0.986	0.981

Standard errors in brackets

* p<0.05,

** p<0.01,

*** p<0.001

### 6.2 Restructuring of elements

The growth of the economy and the efficiency of production may be helped along by a variety of factor inputs, such as money and land. The term "restructuring factors" refers to a collection of many different but connected features that, when combined, have the ability to enhance resource allocation and boost economic growth. Using the study conducted by Yihong et al. (2019) [30], we will now examine the effect that HSR will have on the reorganization of factor markets. Next, we investigate whether or not there is a distinction between the impact of the introduction of HSR on the income of rural residents before and after accounting for the factors that determine fixed asset investment, fiscal spending, education expenditure, and foreign trade. Specifically, we look at whether or not there is a difference between the two scenarios. In accordance with the regression results, the coefficient of the HSR on the income of rural residents is -0.0193 before controlling for these variables and -0.00954 after monitoring for these variables (Table 11), indicating that the consequence of the damaging influence of HSR on the earnings of rural inhabitants becomes weaker after controlling for these variables, thereby increasing the relative income of rural dwellers. Consequently, factor rearrangement may be one of the means via which the introduction of HSR reduces the income of farmers.

**Table 11:** Regression results on the urban-rural income gap after controlling for various factors for the introduction of HSR.

	(1)	(2)	(3)	(4)
HSR	-0.0163***	-0.0134***	-0.0147***	-0.00954*
	[0.004]	[0.004]	[0.004]	[0.004]
FDI	0.0975***	0.0749***	0.0719***	0.0468***
	[0.004]	[0.005]	[0.005]	[0.007]
Expenditure		0.0966***	0.0803***	0.0619***
		[0.012]	[0.013]	[0.014]
Education			0.0396***	0.0259**
			[0.010]	[0.009]
Open				0.126***
				[0.026]
Control variables	be	be	be	be
_cons	7.343***	6.330***	6.114***	5.648***
	[0.065]	[0.146]	[0.145]	[0.146]
Individual Effect	control	control	control	control
Time Effect	control	control	control	control
N	4275	4275	4275	4275
adj.R-sq	0.986	0.987	0.987	0.988

Standard errors in brackets

* p<0.05,

** p<0.01,

*** p<0.001

### 6.3 Labor and capital flows

Based on Fenglong et al. (2018) [31], in this section we recalculate the impacts of the introduction of HSR on labor and capital mobility as well as these two mobilities on the urban-rural income gap. Capital mobility is assessed by fixed asset investment, whereas labor mobility is measured by the number of urban inhabitants and the employed labor force. The results of the HSR labor force shift regression is depicted in columns (1) and (2) of the table below (Table 12), while the results of the HSR capital flows regression are displayed in column (3). On the basis of the results of the regression, it can be stated that the introduction of HSR increases labor mobility and leads to the urbanization of the work force. The correlation of the introduction of HSR with urban resident employment and population is likewise positive and over the 1% significance level. The coefficient of HSR’s influence on investment in fixed assets, which is 0.0371 and statically relevant at the 10% level, demonstrates how the introduction of HSR benefits fixed asset investments. The development of HSR, as stated in this study, has facilitated the transportation of individuals and goods between cities, which has resulted in uneven income growth among urban and rural populations and, as a result, a widening of the income gap between them.

**Table 12:** Impacts of HSR on labor and capital.

	(1)	(2)	(3)
	Number of permanent residents	Number of employed persons	Fixed asset investment
HSR	0.0114***	0.0461***	0.0371*
	[0.003]	[0.011]	[0.017]
Control variables	control	control	control
_cons	15.29***	11.09***	5.154***
	[0.102]	[0.610]	[1.420]
Individual Effect	control	control	control
Time Effect	control	control	control
N	4275	4275	4275
adj.R-sq	0.995	0.938	0.947

Standard errors in brackets

* p<0.05,

** p<0.01,

*** p<0.001

## 7 Discussion and conclusion

### 7.1 Conclusion

Due to China’s transportation infrastructure being in constant development, in this research I employ the DID approach to match panel data from 285 prefecture-level cities over a 15-year period in order to evaluate the effect of the introduction of HSR on the income disparity between urban and rural residents. First, the introduction of HSR has expanded the income difference between urban and rural areas. The results demonstrate that the burgeoning effect of HSR on the urban-rural income gap is still significant even after the robustness test with all explanatory factors behind by one stage, special samples such as eradicating province capital and vice-provincial capital cities, and a placebo test. Furthermore, the wealth disparity between urban and rural areas in the eastern and western regions has grown as a result of the development of HSR, which has exacerbated regional heterogeneity. This has a reversing effect on the urban-rural income disparity in the central area, slightly reducing the imbalance. The analysis of Model 2 demonstrates that whilst the construction of HSR increases urban inhabitants’ income growth, it restrains that of people living in rural areas. By investigating the mechanism of the widening income disparity between urban and rural areas, I have shown that the primary drivers of the widening income gap are the declining incomes of rural people and the migration of labor, money, and other resources between cities.

### 7.2 Recommendations

In recent years, both urban and rural inhabitants’ incomes have improved as a result of China’s strengthening economic position. Due to national policy support, the income of rural inhabitants is rapidly increasing and the income difference between urban and rural areas is decreasing; nonetheless, the issues brought about by the significant income gap between urban and rural areas cannot be disregarded. In terms of justice, people’s happiness, economic growth, and social stability, closing the income gap between urban and rural areas and raising the income of rural residents remain critical issues. Consequently, we make the following recommendations:

(1) Make the network of urban-rural transportation more accessible and well-organized.

In recent years, as a result of the high level of urban development and economic strength, the transport infrastructure has become relatively more complete. However, in some rural and remote areas, due to the lack of construction funds, terrain that is rugged and difficult to access, and other factors, the construction of transport infrastructure is still underdeveloped, and is far from being able to meet the development of modern agriculture, modern industry, and trade and logistics. This is not only reflected in the backwardness of the intra-countryside transport network, but also in the fact that the construction of the transport network between the countryside and the city is relatively weak, which restricts the development of rural areas and the increase in the income of farmers. The construction of a convenient transport network between urban and rural areas will strengthen economic exchange between these areas, drive rural development in urban areas, and promote the integrated construction of urban and rural areas. The most important aspect of integrated urban-rural development is the transport connection between urban and rural areas, which would enable the full circulation of people and goods. Urban-rural transport integration can be used to crack the urban-rural transport dichotomy; comprehensively promote urban-rural integration; serve the economic and social development between urban and rural areas with the transformational development of transport; serve new urbanization and new rural construction; and focus on the planning layout of central towns and villages. An acceleration of transport service support for the development of HSR would tilt high-quality transport resources towards the majority of rural areas, enabling rural residents to enjoy the benefits of convenient travelling and promote the smooth flow of rural agricultural products into the city, so rural residents can benefit from the development.

(2) Create prosperous HSR-based enterprises that provide job opportunities for rural populations.

With the development of HSR, its coverage is getting wider and wider, and the radiation range is getting bigger and bigger, which has an important impact on the production and lives of common people. Compared with other means of transport, HSR is faster, safer, and more reliable, and is less affected by the weather, and can thus enhance people’s travelling experience and efficiency. At the same time, the economic development of a place is closely related to the convenience of local transport and the degree of coverage of the transport network, and the construction and operation of HSR can stimulate the regional economic development along the route, promote industrial agglomeration and transformation and upgrading of industrial infrastructure, and bring into play the effect of economies of scale of enterprises. The full-scale development of HSR is bound to promote the development of related industries. The construction of HSR needs investment in a lot of manpower, materials, and capital, and there will be a great demand for cement, iron, and steel, construction machinery, and goods and services from other industries, through which HSR can effectively stimulate the upgrading of technology and equipment in HSR-related industries and promote the optimization and upgrading of entire industries, so as to create more employment opportunities.

(3) Strengthen the market mechanisms between urban and rural areas to encourage the sensible movement of factors (such as labor and capital) between urban and rural areas and regions.

From the mechanism study above, we know that the introduction of HSR is conducive to the free flow of labor, capital, and other factors between urban and rural areas, but this transfer has not led to the alleviation of the urban-rural income gap, and the cause of this phenomenon may be due to irrational and uncoordinated flows. The location of HSR stations is closely related to the economic activity and population density of a city; the economic effect of HSR will firstly be reflected in the regional central city, due to the relative development of the central city, then under the market mechanism, the labor force will be transferred from the general cities and rural areas to the more economically developed cities, and the general cities and rural areas will face population loss. Likewise, resources such as capital and other factors that are profit-seeking by nature will also be concentrated in the big cities, and a situation of the strongest getting stronger will emerge. In this regard, on the one hand, it is necessary to actively expand the scope of influence of HSR, so as to allocate resources more widely and improve the radiation scope of HSR construction to urban and rural areas; on the other hand, the rapid flow of resources brought about by HSR requires greater administrative capacity and higher government efficiency. Therefore, in order to satisfy the demand for linked economic development brought about by HSR, it is necessary for government to continuously improve its efficiency, improve and optimize the level of management and services, and focus on guiding the reasonable flow of labor resources and capital, paying more attention to policy and financial support for small and medium-sized cities and rural areas, and introducing more positive and reasonable policies to serve economic development, such as attracting and encouraging talents to participate in the economic construction in the vast rural areas, providing talents and technical support for the development of the rural industry, introducing locally-adapted enterprises and industries for rural areas, and absorbing the idle labor force in rural areas to increase farmers’ additional income.

(1) Increase funding to the west and promote balanced regional development.

Modern economy is closely related to the progress of means of transport. The development of HSR is profoundly changing the economic map of our country. The construction of HSR in the eastern, central, and western regions is unbalanced, due to the huge land mass and the better economic development of the east and central parts of the country. The construction of HSR in these two regions started early and with stations covering a wide range. On the other hand, in the western part of the country, which is a less economically advanced region, construction of HSR started late. At present, the backbone routes of China’s HSR are mainly laid out in the eastern part of the country, and the density and technical standard of HSR in the western part of the country are relatively low. However, from the perspective of future development changes and the overall economic and social situation, to co-ordinate urban and rural areas and regional development, and to narrow the income disparity between the regions and the urban and rural areas, it is even more necessary to speed up the construction of HSR in the western part of China. The western region is located inland, but with a vast land area, transport is an important link to realize the economic take-off of the west and strengthen the connection with the mainland. The western region has to build a modern transport system in order to narrow the development gap with the eastern region. In the long run, the construction of HSR in the western region will not only protect the ecological environment, but also enhance national unity and promote two-way population flow. To accelerate the construction of HSR in the western region, it is necessary to strengthen the construction of the corridors between the west and the east and to strengthen the construction of the east-west corridors, and, while focusing on the extent of HSR construction, attention should also be paid to improving the speed and capacity of HSR in the west.

(2) Locations for HSR stations should be chosen reasonably, and the price of public HSR tickets should be kept to a minimum.

The location of HSR stations is determined after weighing a variety of complex factors such as the flow of people, the economic level of the city, etc. In order to give the HSR project a wider scope of influence, the location of HSR stations should be planned reasonably in order to promote the integrated development of urban and rural areas, which not only ensures the accessibility of the HSR, but also serves the development of the local area, and reduces the segmentation of the city as far as possible.

HSR ticket prices are relatively high compared to ordinary train ticket prices, not only because of HSR’s speed and convenience, but also because of the large investment in the preliminary construction of HSR and high maintenance costs. At the same time, HSR promotes the flow of labor and capital between urban and rural areas, and has an important role in increasing the income of urban and rural residents. In order to further enhance HSR services for the general public, especially for the peasant masses, it is necessary to set reasonable HSR fares, so as to make them more acceptable and affordable for the general public, thus enhancing the tendency to choose HSR as their preferred means of transport.

The information in this paper is only a phasic result of the author’s investigation into "the effect of HSR on the economic gap between urban and rural inhabitants," and there are several related issues that need more research. More thorough data should first be acquired. There are also some missing urban statistics. Future studies can be more convincing and thorough by evaluating city data at the county level due to the limitations of present city samples at the prefecture level. Also, there was no dynamic inquiry. The HSR’s introduction is not a one-time, nationwide event. This study neither examined nor distinguished between the dynamic impact and lag effect of HSRs every year after introduction, nor did it provide an explanation for the lag effect. Thirdly, the factors mentioned are also influenced by other modes of transportation, including conventional trains and cars. Future studies should examine the effects of new modes of transportation on the variables that have been stated or include more modes of transportation for comparative analysis.

## Supporting information

S1 File (XLSX)
